# A Comparative Study on Synthesizing SiC via Carbonization of Si (001) and Si (111) Substrates by Chemical Vapor Deposition

**DOI:** 10.3390/ma18143239

**Published:** 2025-07-09

**Authors:** Teodor Milenov, Ivalina Avramova, Vladimir Mehandziev, Ivan Zahariev, Georgi Avdeev, Daniela Karashanova, Biliana Georgieva, Blagoy Blagoev, Kiril Kirilov, Peter Rafailov, Stefan Kolev, Dimitar Dimov, Desislava Karaivanova, Dobromir Kalchevski, Dimitar Trifonov, Ivan Grozev, Valentin Popov

**Affiliations:** 1 ”Acad. E. Djakov” Institute of Electronics, Bulgarian Academy of Sciences, 1784 Sofia, Bulgaria; tmilenov@ie.bas.bg (T.M.); skkolev@ie.bas.bg (S.K.); dadimov@ie.bas.bg (D.D.); dkaraivanova@ie.bas.bg (D.K.); dtrifonov@ie.bas.bg (D.T.);; 2 Institute of General and Inorganic Chemistry, Bulgarian Academy of Sciences, 1113 Sofia, Bulgaria; 3 ”G. Nadjakov” Institute of Solid State Physics, Bulgarian Academy of Sciences, 1113 Sofia, Bulgaria; vmehandzhiev@issp.bas.bg (V.M.); blago@issp.bas.bg (B.B.); rafailov@issp.bas.bg (P.R.); 4 Institute of Physical Chemistry “Rostislaw Kaishev”, Bulgarian Academy of Sciences, 1113 Sofia, Bulgaria; izahariev@ipc.bas.bg (I.Z.); g_avdeev@ipc.bas.bg (G.A.); 5 Institute of Optical Materials and Technologies “Acad. Jordan Malinowski”, Bulgarian Academy of Sciences, 1113 Sofia, Bulgaria; adi@iomt.bas.bg (D.K.); biliana@iomt.bas.bg (B.G.); 6 Faculty of Physics, Sofia University “St. Kliment Ohridski”, 1164 Sofia, Bulgaria; kirilowk@phys.uni-sofia.bg (K.K.); vpopov@phys.uni-sofia.bg (V.P.); 7 National Centre of Excellence Mechatronics and Clean Technologies, Bulgarian Academy of Sciences, 8 Kliment Ohridski Blvd., Blk. 8, 1756 Sofia, Bulgaria

**Keywords:** silicon carbide, chemical vapor deposition, powder X-ray diffraction, X-ray photoelectron spectroscopy, transmission electron microscopy

## Abstract

This work presents a comparative analysis of the results of silicon carbide synthesis through the carbonization of Si (001) and Si (111) substrates in the temperature range 1130–1140 °C. The synthesis involved chemical vapor deposition utilizing thermally stimulated methane reduction in a hydrogen gas stream. The experiments employed an Oxford Nanofab Plasmalab System 100 apparatus on substrates from which the native oxide was removed according to established protocols. To minimize random experimental variations (e.g., deviations from set parameters), short synthesis durations of 3 and 5 min were analyzed. The resultant thin films underwent evaluations through several techniques, including X-ray photoelectron spectroscopy, X-ray diffractometry, optical emission spectroscopy with glow discharge, and transmission electron microscopy. A comparison and analysis were conducted between the results from both substrate orientations.

## 1. Introduction

Silicon carbide (SiC) is a wide-bandgap semiconductor well-regarded for its excellent thermal conductivity, considerable breakdown electric field, and high mechanical strength, making it ideal for high-power, high-frequency, and high-temperature electronic applications such as power metal–oxide–semiconductor field-effect transistors, Schottky diodes, and RF components. SiC’s chemical stability and thermal durability also render it useful in harsh environment sensors and protective coatings [1]. Additionally, many applications of this material were developed for nuclear energy [2,3], photocatalysis [4], epitaxial synthesis of graphene [5], and gas-tight SiC coatings to protect carbon fibers/nanotubes from oxidation, ceramic parts from harmful gas effects, etc. [6,7]. The synthesis of low-dimensional forms of SiC, e.g., 1D [8,9,10,11] and 2D/quasi-2D [12], has also been identified as an area of growing interest in recent years.

Despite the availability of bulk SiC wafers, their high cost and limited size pose obstacles to large-scale device integration. Therefore, the heteroepitaxial growth of SiC thin films on more affordable and commonly used silicon substrates presents a viable technological solution. Such growth has been proposed by [13] via chemical vapor deposition (CVD). This method is particularly valued for its ability to create high-quality, high-purity, uniform, and large-area SiC films [14]. Variations in numerous parameters such as the hydrocarbon precursor, carrier gas, and system pressure are permissible in CVD processes. However, growing SiC on Si substrates remains technically challenging due to the substantial lattice mismatch and varying thermal expansion coefficients between Si and SiC, which frequently results in high densities of structural defects, including stacking faults, threading dislocations, and cracks [15]. Two established methods aim to reduce these defects: the first involves a carbonized buffer layer formed by exposing the Si surface to a carbon-containing gas at temperatures above 1200 °C before SiC growth. An alternative, simpler method is direct carbonization, which creates a SiC layer directly on the Si surface without an intervening buffer layer [16].

Notable advancements in the heteroepitaxial growth of high-quality SiC have been made by Nishino et al. [17] and Severino et al. [18]. The former introduced a two-step growth method featuring a carbonization step followed by high-temperature epitaxial growth, which helps enhance nucleation density and improve surface morphology [17]. The latter method involves two additional processes: high-temperature etching in a reductive gas flow and the deposition of a thin protective carbon film at temperatures lower than the carbonization temperature [18]. Specifically, the synthesis of intermediate thin films from SiC at temperatures of 1020–1300 °C for further deposition of CVD layers from SiC for electronics/microelectronics was also considered at that time [19,20]. The authors of these works, however, characterized the films with local methods of analysis (scanning electron microscopy (SEM) and transmission electron microscopy (TEM)), and this limited their results. In the last few years, research on CVD synthesis of SiC has focused mainly on the high-temperature epitaxial growth of SiC, in search of alternative precursors [21] and lowering the temperature of this reaction from above 1400 °C down to 1120 °C [22].

Recent studies have also investigated the synthesis of an SiC intermediate film on Si (111) substrates at a lower temperature of 1140 °C, considering the impact of native oxide on the resultant films through theoretical molecular dynamics methods [23] and experimental CVD on Si (111) substrates via the reduction of CH_4_ precursor in a H_2_ main gas flow [24]. The former study determined that the presence of native oxide does not impede the process, and carbon atoms enter the Si wafer only after complete hydrogen loss. High temperatures ensure that the crystal surface maintains a state of reconstruction, negating the need for substitution reactions for the incorporation of C/C–O complexes onto the Si substrate [23]. The latter study’s experimental results suggest SiC is synthesized in nearly all cases, irrespective of the native oxide cleaning and synthesis time, showing no clear evidence of a distinct carbon phase on the thin films. Additionally, the thin films lack a defined interface with the substrates; due to slab surface reconstruction at this temperature, Si–C and Si–O–C complexes readily penetrate the Si matrix, forming a thicker area layer that is similar in thickness to the SiC layer [24].

This work compares the synthesis of SiC layers on Si (001) and Si (111) substrates in a single-step CVD process through direct carbonization. Our purpose was to investigate the possibilities for the synthesis of intermediate thin SiC films at temperatures up to 1150 °C, i.e., temperatures compatible with silicon technology, for further epitaxial deposition of SiC layers. By examining the crystalline structure, phase composition, and interface characteristics, we analyze the impact of substrate orientation on SiC nucleation and growth. Our findings enhance the understanding of SiC heteroepitaxy and offer guidance for the future integration of SiC films into silicon-based technologies.

## 2. Materials and Methods

One-sided-polished 3-inch diameter Si (001) as well as Si (111) wafers (Wacker Chemie, Munich, Germany) were used in all experiments. The precursors for all processes were N_2_ (99.999% purity), H_2_ (99.999% purity), Ar (99.99% purity), and CH_4_ (99.9995% purity) (Linde Gas Bulgaria, Sofia, Bulgaria). Carbonization processes were carried out using CVD in an Oxford Nanofab Plasmalab System 100 apparatus (Oxford Instruments, Abingdon, UK). All experiments were performed on substrates from which the native oxide was removed according to established protocols [25,26]. To verify the reproducibility of the CVD processes, they were repeated two to three times with consistent results. To further reduce the native oxide levels in all experiments, substrates were cleaned in Ar plasma at a temperature of 250 °C (Ar flow of 500 sccm, pressure of 3000 mTorr, power of 50 W, and mid-frequency plasma at 13.56 MHz, with a 10 min plasma etching time). Subsequently, all substrates were annealed in an atmosphere of H_2_ (500 sccm, pressure of 200 mTorr) within the temperature range 1130–1140 °C for 5 min before undergoing the direct carbonization processes on the Si surface.

All thermal treatments occurred in a vacuum (thermal annealing before the carbonization processes) of 200 or 300 mTorr (including heating, cooling, and carbonization). The heating was conducted at a rate of approximately 600 °C/h with a 10 min pause at 250 °C (for Ar plasma etching) in all cases. The carbonization was achieved through thermally stimulated CVD for the decomposition of CH_4_ at 1130–1140 °C and 300 mTorr in an H_2_ (500 sccm) and carbon-containing precursor (10 sccm) gas mixture with a duration of 3 and 5 min. Cooling to room temperature after the carbonization processes occurred in a gentle flow of Ar and H_2_, complemented by natural reactor cooling over a period of approximately 15 to 18 h.

X-ray Photoelectron Spectroscopy (XPS) was used to characterize the surface composition, employing a VG ESCALAB MK II electron spectrometer (Thermo Fisher Scientific, Waltham, MA, USA) with a non-monochromatized Al Kα radiation source (photon energy of 1486.6 eV). Measurements were conducted under ultra-high-vacuum conditions, maintaining a base pressure of 1 × 10^−8^ Pa. Instrumental energy resolution was evaluated via the Ag 3d_5_/_2_ core level at analyzer pass energy of 20 eV, yielding a full width at half maximum of 1 eV. Spectrometer calibration was completed using the Au 4f_7_/_2_ reference peak (84.0 eV), and surface-charging effects were corrected based on the C 1s signal at 285.0 eV, which is attributed to naturally occurring hydrocarbon contaminants. Binding energy accuracy was estimated to be within ±0.2 eV.

Core-level spectra corresponding to C 1s, O 1s, Si 2p, and N 1s were recorded along with minor signals from surface contaminants like F 1s and Cu 2p. All spectra underwent background subtraction (Shirley-type) and quantification via peak areas adjusted by Scofield photoionization cross-sections. Peak deconvolution and fitting processes utilized the XPSPEAK4.1 software package.

The thickness of the specimens was determined by ellipsometry measurements, which were performed using a Woollam M2000D (J.A. Woolam Co. Inc., Lincoln, NE, USA) rotating compensator spectroscopic ellipsometer with a wavelength ranging from 193 to 1000 nm. The data acquisition and modeling software was CompleteEASE v.5.19 (J.A. Woollam Co., Inc.).

To comprehensively analyze the in-depth elemental distribution in thin films, two complementary techniques were employed: argon ion sputtering combined with glow discharge optical emission spectroscopy (GDOES) with a GDA 750 HR spectrometer (Spectruma Analytik GmbH, Hof, Germany). Powder XRD measurements were conducted using a Philips PW 1050 diffractometer (Koninklijke Philips N.V., Eindhoven, The Netherlands) equipped with a Cu Kα tube at 18 °C. High-resolution transmission electron microscopy (HR TEM) and selected area electron diffraction (SAED) were performed on an HR STEM JEOL JEM 2100 (Jeol Ltd., Akishima, Tokyo, Japan) microscope at an accelerating voltage of 200 kV.

## 3. Results

Our earlier research [10] demonstrated that trapping of C–O/C–O–C radicals on the Si substrate surface and the diffusion of C and O atoms into the substrate created a poorly defined interface between the substrate and the thin film, complicating thickness measurements in Ultra-High-Strength Steel (UHSS) blade-scratched areas.

The thicknesses of the synthesized thin films C3_001, C5_001, C3_111, and C5_111 were determined by ellipsometric measurements, and were 12, 13, 11.9, and 14 nm, respectively. To maximize the accuracy of the fits, we used the results from the XPS and GDOES studies on the film compositions as input parameters. These were approximated with layers containing 4H–SiC and Si–C–O glass, assuming there is no discrete SiO_(2−x)_ phase.

It is widely recognized that XPS provides accurate information on chemical composition and bonding states within about 15 nm of the thin film surface. In this study, XPS was employed to evaluate the surface concentrations of the constituent elements and to identify the chemical bonding configurations in the deposited thin films. The quantified surface compositions are summarized in Table 1, which includes comparative data for samples synthesized under similar conditions on Si (111) substrates, as previously reported in Ref. [24].

**Figure 1:**
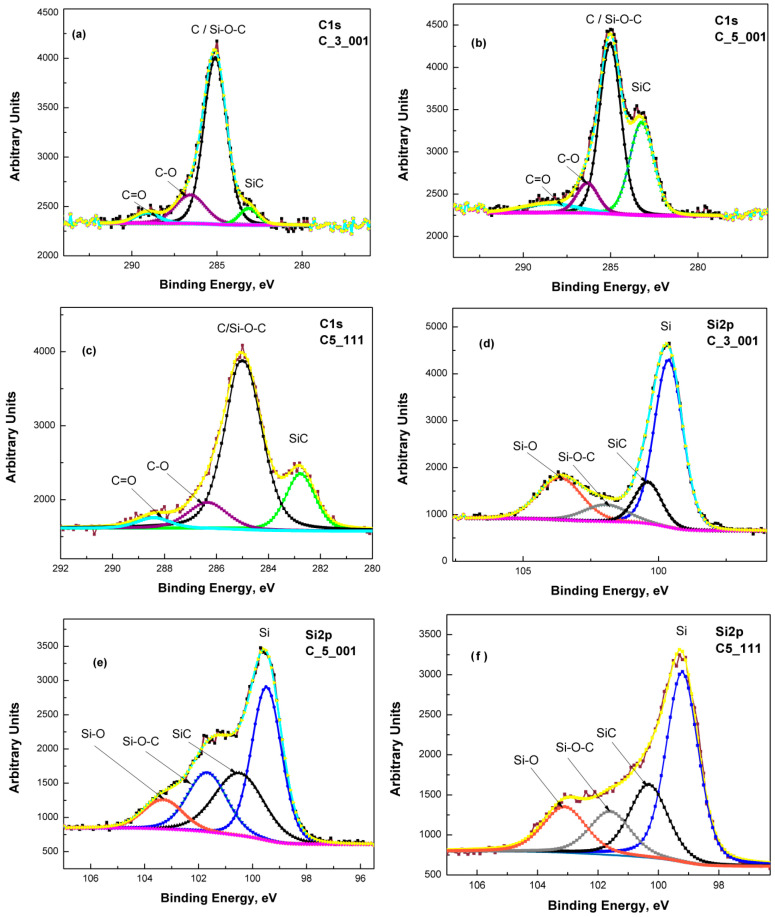
Deconvolution of the C 1s line of samples: C3_001 (**a**), C5_001 (**b**), and C5_111 (**c**): the green trace corresponds to SiC, while black, violet and cyan ones to C/Si-O-C, C-O and C=O, respectively. Deconvolution of the Si 2p line of samples: C3_001 (**d**), C5_001 (**e**), and C5_111 (**f**): the blue trace corresponds to Si, while black, grey and orange ones to SiC, Si-O-C and Si-O, respectively.

For this study’s objectives, particular attention was given to analyzing the C 1s and Si 2p core-level spectral regions. The deconvoluted XPS spectra for these core levels are displayed in Figure 1a–f. The C 1s spectra revealed four distinct components associated with specific chemical environments (Figure 1a–c): C–Si bonds (indicative of SiC and silicon oxycarbide phases) around 283.2 eV, C–C (graphitic or amorphous carbon) around 285.0 eV, and oxygen-containing carbon species (C–O and C=O) at 286–287 eV and above 288–289 eV, respectively. These assignments align with the NIST XPS database [27]. The Si 2p spectra also resolved into multiple components, corresponding to various Si bonding states (Figure 1d–f). Four major contributions were detected: elemental silicon (Si–Si) at 99.8 eV, silicon carbide (Si–C) at 100.2 eV, silicon oxycarbide (Si–O–C) at 101.7 eV, and silicon dioxide (Si–O) at 103.8 eV. These binding energy values correlate well with previous research findings regarding stoichiometric Si, SiC, and SiO_2_ phases (e.g., Önneby and Pantano [28]).

**Figure 2:**
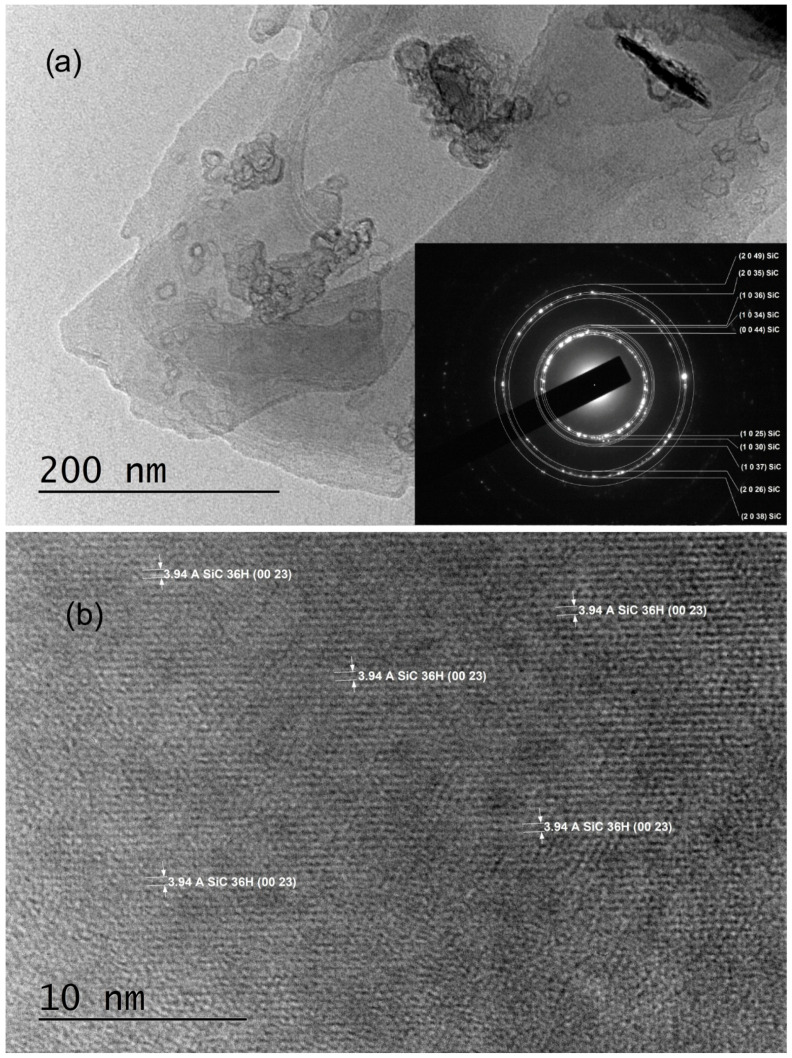
TEM images obtained from C3_001 samples: (**a**) A low-magnification TEM image along with the corresponding SAED image; (**b**) an HRTEM image taken from an area near to the most transparent region of the crystal flake shown in panel (**a**).

**Figure 3:**
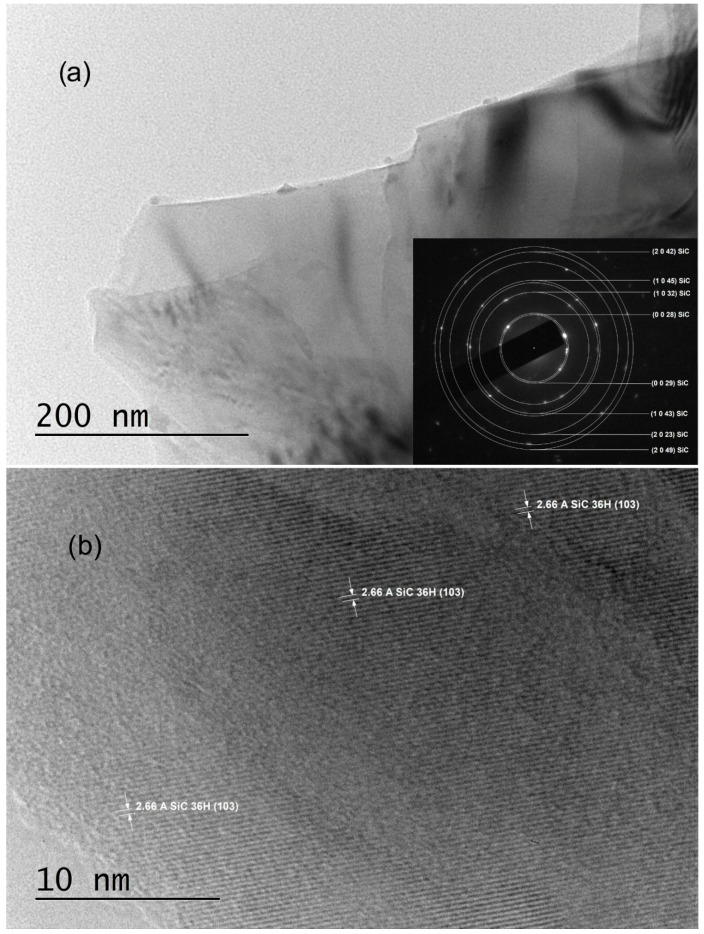
TEM images obtained from C5_001 samples: (**a**) A low-magnification TEM image along with the corresponding SAED image; (**b**) an HRTEM image taken from an area near to the most transparent region of the crystal flake shown in panel (**a**).

The TEM sample preparation process involved mechanically scratching their surfaces with a UHSS blade. Characterization via SAED revealed polycrystalline structures indexed solely with trigonal symmetry (SG #156 and cell parameters a = 3.0730 Å, c = 90.6500 Å), specifically as 36H–SiC (shown in the insets of Figure 2a and Figure 3a, as well as in Ref. [29]). Notably, the SAED patterns commonly exhibited polycrystalline characteristics, with bright crystalline reflections in nearly all samples indicating well-ordered crystallite structures.

Various crystal planes are discernible in HRTEM images (Figure 2b and Figure 3b); the crystal planes (0023) of 36H–SiC are evident in the entire field of Figure 2b, with noticeable, extensive two-dimensional crystal plane networks. In the HRTEM image of Figure 3b, the (103) crystal planes of 36H–SiC are also visible across most of the field.

**Figure 4:**
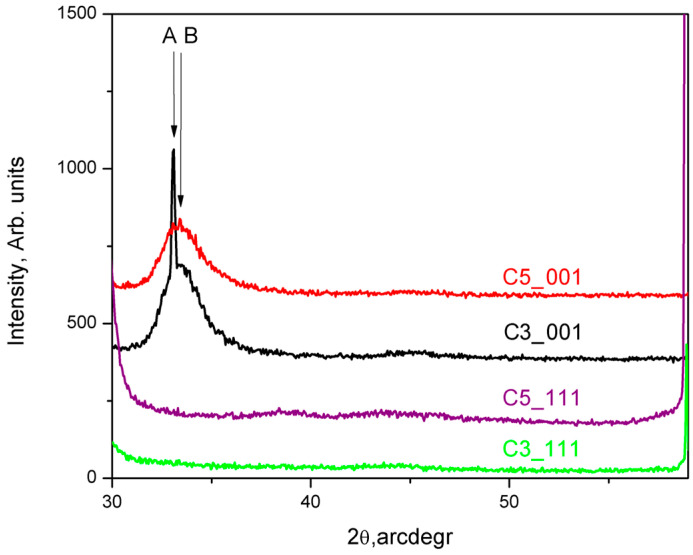
XRD patterns taken from the C3_001, C5_001, C3_111, and C5_111 samples are shown in black, red, violet, and green traces, respectively.

XRD patterns for the obtained thin films were measured using (θ–2θ) scans over the range of 2θ = (30–60)° with step-scan mode at increments of 0.050 and 3 s/step for XRD data collection. The results for the samples C3_001 and C5_001 are presented in Figure 4 with black and red traces, respectively. XRD patterns were measured under the same experimental conditions for films grown on perfectly cleaned and oxide-free Si (111) substrates, as reported in our previous research [24] (Figure 4). While XRD pattern data alone do not allow precise indexing of the diffractograms, assuming the structure indexing of the thin films to be 36H–SiC, based on the findings of the SAED studies (see above), permits some reasonable assumptions. Both XRD patterns of thin films deposited on Si (001) substrates are marked by a notably broadened diffraction peak around 2θ = 33.2°, marked as B in the red and black traces in Figure 4. This peak, located within the range 2θ = (32.6–36.0)°, cannot be precisely identified. Additionally, a very sharp and significantly more intense peak, labeled A in Figure 4, observed at 2θ = (33.5–33.6)° in some XRD patterns, can be assigned to d_(00–34)_.

Furthermore, we analyze the peak profiles of the XRD patterns of C3_001 and C5_001 samples by their deconvolution into Gaussian and Lorentzian components, from which the values for crystallite sizes and microstresses were calculated (see Ref. [30]). Our calculations show that the crystallite sizes for the diffuse peaks denoted by B of the red and black traces in Figure 4 at 2θ = 33.7–33.8° are about 2.9 nm. The calculated microstrain is negative, approximately equal to −0.1%. We suggest that since the deposited thin film/s is/are very thin and strongly influenced by the substrate, compressive stresses are generated in the first few nanometers. The narrow peak at 2θ = (33.5–33.6)° has a crystallite size of about 88 nm and positive microstrain. Therefore, we attribute the observed changes in the shape and position of the XRD peaks mainly to the small grain size and the existing microstrains; however, the presence of structural defects such as stacking folds and twin lamellae (see Ungar [31]), which enhance these effects, should also be considered.

**Figure 5:**
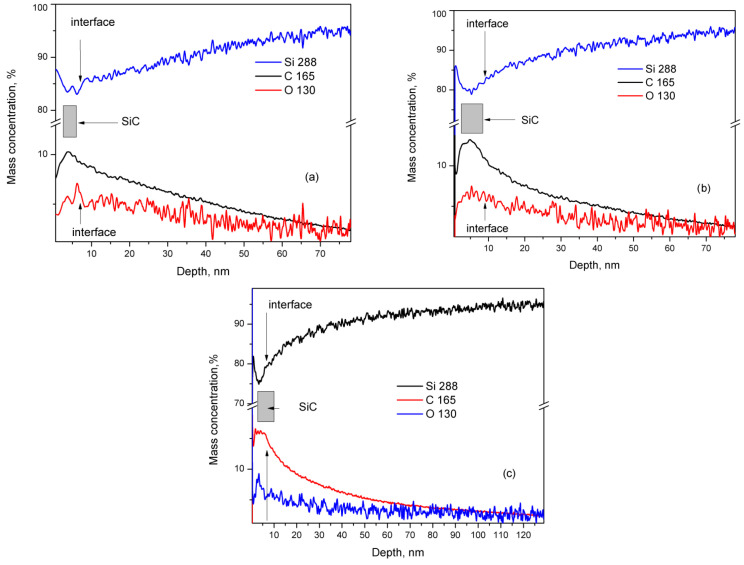
GDOES study of samples: (**a**) C3_001, (**b**) C5_001, and (**c**) C5_111. The mass concentration of Si is drawn by blue traces, while those of C, O, and N are drawn by black, red, and violet traces, respectively.

GDOES is employed to analyze the elemental depth distribution of silicon, carbon, and oxygen in the thin films, using a Spectrum Analytic GDA 750 HR instrument (Spectruma 199 Analytik GmbH, Hof, Germany). Measurements were conducted in RF mode under constant voltage and controlled pressure, with Si–C optimized settings at 800 V and 3 hPa. Prior to analysis, samples underwent a 30 s evacuation and a subsequent 30 s exposure to high-purity Ar gas. Representative depth profiles for select samples are displayed in Figure 5a,b. It is crucial to acknowledge that plasma-assisted material removal during sputtering favors the ejection of silicon atoms from SiC-containing layers, leading to carbon underrepresentation in depth profiles [32]. Additionally, GDOES measurements help determine the thin film thickness, as detailed in our previous study on SiC synthesis on Si (111) substrates [24]. In this context, we assert that the most active SiC synthesis occurs at the thin film–substrate interface, inferring maximum carbon concentration (± a few percent) within the film. Consequently, we estimate thin film thicknesses of approximately 5–6 nm for C3_001 samples and 7–8 nm for C5_001 samples. The GDOES results for sample C5_111 from Si (001) substrate resemble those from Si (001) substrates, particularly C5_001 (Figure 5b). Nevertheless, the SiC thin film thickness for C5_111 of around 6 nm indicates an approximately 15% reduction compared to C5_001, with a significantly larger diffusion depth of C and O into the substrate of around 100 nm.

## 4. Discussion

The characterization of 5–10 nm thick SiC thin films synthesized on Si substrates, consisting of SiC and Si–O–C glasses, presents several challenges. The non-destructive XPS study provides insights into the phase composition, based on binding energies of the detected elements, only for the top 15 nm and averaged across the entire studied volume. Conversely, in-depth XPS analysis, combined with sequential magnetron sputtering of the film’s surface, offers a clear assessment of the phase composition of the studied objects; however, this approach lacks assurance that the sputtered phase matches the solid phase composition. TEM, SAED, and HRTEM analyses yield precise but localized results, with potential uncertainties when examining materials containing substantial amorphous phases. Powder and Grazing Incidence XRD are vital for analyzing crystalline phases. Raman and IR spectroscopies encounter limitations when investigating thin films with intricate structures. Hence, a carefully selected set of analytical techniques must be employed for compositional analysis, while the interpretation should integrate all these results. For ensuring the reliability of the characterization results, we conducted the following studies: (1) XRD studies, in which each of the 3-inch samples was measured at five points, one approximately at the center of the plate, and the remaining ones symmetrically positioned on a circle with a radius equal to half that of the plate; the so-measured XRD patterns were representative of the entire sample. (2) XPS studies, in which 2–3 pieces with an area of about 5 × 5 mm^2^ from each sample were analyzed; when analyzing the results, it was taken into account that the signal was collected from about a 15 nm depth in the samples, i.e., the determined concentrations were averaged over the entire studied area. (3) GDOES studies, in which 2–3 pieces from each sample were analyzed; the analyzed area was chosen as a circle with a diameter of about 7.5–8.0 mm. Our results show the distribution of mass concentration in the depth of the sample and provide the necessary “correction” when jointly analyzing the XPS and GDOES data. The characterization of the samples within the specified framework provides reliable results for the samples at the macro scale.

The above conclusions are supported by the fact that the thickness of the thin films determined by ellipsometric measurements is between 50 and 100% greater than that determined by GDOES measurements. As remarked in our recent publication [24], as a result of the enrichment of the Si-substrate surface area by O and C just below the substrate/thin film interface, the optical characteristics of the substrate in this area change and create prerequisites for inaccuracy in the fits of the data from the ellipsometric measurements.

TEM and SAED analyses of samples C3_001 and C5_001 (insets of Figure 2a and Figure 3a) confirm the crystallite structure of thin films to be 36H–SiC, with no alternate crystalline phases detected in any examined samples. The observation of a single broadened peak within the XRD spectra (B in Figure 4) indicates the presence of an ordered crystalline phase with highly aligned crystallites along the <001> direction. This peak could be caused by a superposition of numerous high-intensity reflections in the 2θ = (32–36)° region, such as d_(013)_, d_(1–13)_, d_(−113)_, d_(014)_, d_(1–14)_, d_(−114)_, etc. [15]. However, this interpretation remains tenuous due to the absence of discernible reflexes at alternate 2θ values.

Determining the precise phase composition of these thin films remains a considerable challenge. Although the existence of SiC, Si–O–C groups/species, and minor quantities of Si–O, Si=O, C–O, and C=O radicals can be established, quantitatively assessing ratios like Si/SiC and SiC/Si–O–C is a process with significant obstacles. For instance, analyzing a 5–7 nm thick film yields XPS signals collected from the substrate, approximating 50–70% of the main thin film volume, with varying percentages for different materials. Consequently, the obtained results for the SiC/C (Si–O–C) ratio from the deconvolution of the C 1s line in the C3_001 sample (Figure 1a and Table 2) appear anomalously low.

However, variations in composition across samples are possible, as the films were produced on 3-inch Si wafers while XPS analysis focused on about a 5 × 5 mm^2^ area. Additionally, the Si–O–C species signal may partially mask the pure SiC signal in the C 1s line region (Figure 1a and Table 2). Nevertheless, the GDOES findings maintain relative accuracy regarding thin film thickness, as discussed in our earlier research [10]. We can reliably assume the thickness of thin films from C3_001 experiments to be 5–6 nm, with C5_001 roughly measuring 7–8 nm. It also appears that SiC thin film synthesis on (001) Si substrates occurs predominantly at the interface between the substrate and the created film. Further confirmation of the findings from our prior study [24] indicates that beneath the SiC thin film, the Si substrate shows a region of elevated C and O concentration, typically as C–O radicals. The diffusion of these impurities from the substrate surface into its volume drives its formation, as outlined in Ref. [24]. The concentration of these impurities gradually decreases to a minimum of around 65–70 nm in samples C3_001 and C5_001. The thickness of this elevated concentration layer in C3_001 is slightly reduced compared to C3_111’s 70–75 nm region [24], appearing approximately 10–12% smaller. Conversely, in C5_111, this region measures approximately 100 nm (Figure 5c), nearly double that of C5_001. These insights likely relate to enhanced diffusion rates of O and C in Si substrates along the <111> direction as opposed to the <001> direction, matching previous findings by Dolbak and Olshanetsky (refer to Ref. [33]) regarding Ge diffusion in Si.

Combining the results of XPS and GDOES studies and taking into account that O is incorporated into the SiC lattice as Si–O–C groups, we can reasonably assume that its concentration in SiC thin films is within a few percent, while it decreases to background values in the substrate region below the thin film/substrate interface.

Thus, we deduce that thin films synthesized with this technique could be used as intermediate layers for the subsequent synthesis of thicker SiC layers for microelectronics applications. Additionally, given the production of highly textured SiC thin films, they can be directly applied to create more intricate electronic components, detectors, and others in electronics and microelectronics.

## 5. Conclusions

The results of the synthesis of SiC by direct carbonization of Si (001) substrates are compared with those on Si (111) substrates. Si (001) substrates, cleaned of native oxides according to well-known protocols, were used in the experiments. Carbonization processes were carried out in the Oxford Nanofab Plasmalab System 100 apparatus by thermal reduction of CH_4_ in an Ar flow. The characterization of the resultant thin films employed powder XRD, XPS, TEM/HRTEM/SAED, and GDOES techniques. The findings indicate that crystalline SiC thin films around 5–6 nm and 7–8 nm thick were synthesized for 3 and 5 min of carbonization time, respectively. The synthesized thin films were indexed to trigonal symmetry (SG #156 with cell parameters a = 3.0730 Å and c = 90.6500 Å), constituting 36H–SiC, with pronounced texture along the <001> direction. All SiC thin films contained small amounts of oxygen (up to a few %). For every sample synthesized on the Si (001) substrate, a region was detected at the SiC/substrate interface, where concentrations of C and O gradually decreased from the interface’s level to the minimum consistent with Si (001) substrates. The average size of crystal grains in thin films deposited on Si (001) substrates is about 3 nm, but crystallites with a size of about 90 nm are also observed.

## Figures and Tables

**Table 1:** Chemical composition of the thin films determined by XPS survey studies for samples C3_001 and C5_001.

Sample	C, at.%	O, at.%	N, at.%	Si, at.%
C3_001	18.32	27.30	4.92	49.46
C3_111	37.24	27.69	5.45	29.46
C5_001	32.63	18.09	3.52	45.37
C5_111	31.65	20.18	5.17	42.47

**Table 2:** Percentage content of the different components according to the deconvolution of the C 1s and Si 2p lines of the XPS spectra.

Experiment	C 1s Line				Si 2p Line			
	SiC,%	C/C–Si–O,%	C–O,%	C=O,%	Si,%	SiC,%	Si–O–C,%	Si–O,%
C3_001	6.0	72.7	16.5	4.8	56.1	13.5	8.7	21.7
C3_111	10.7	62.5	19.2	7.5	49.1	14.5	8.3	28.1
C5_001	31.6	52.1	9.1	7.2	41.0	27.7	20.9	10.3
C5_111	18.6	55.1	19.2	7.1	42.8	21.0	18.1	18.1

## Data Availability

The original contributions presented in this study are included in the article. Further inquiries can be directed to the corresponding author.

## Abbreviations

The following abbreviations are used in this manuscript:

CVD	Chemical Vapor Deposition.
XPS	X-ray Photoelectron spectroscopy.
GDOES	Glow Discharge Optical Emission Spectroscopy.
XRD	X-ray Diffraction.
TEM	Transmission Electron Microscopy.
HRTEM	High-Resolution Transmission Electron Microscopy.
SAED	Selected Area Electron Diffraction.
